# Turning any bed into an intensive care unit with the Internet of things and artificial intelligence technology. Presenting the enhanced mechanical ventilator

**DOI:** 10.12688/f1000research.127647.1

**Published:** 2022-12-23

**Authors:** Leidy Lorena Pulido Morales, Juan Sebastian Buitrago Romero, Ismael A. Ardila Sanchez, Fernando Yepes-Calderon

**Affiliations:** 1 Facultad Tecnológica, Universidad Distrital Francisco José de Caldas, Bogota, Bogota, Colombia; 2 I+D+I, GYM Group SA, Cali, Valle del Cauca, Colombia; 3 Research and Development, Science Based Platforms LLC, Fort Pierce, Florida, 34950, USA; 4 Facultad de Medicina, Universidad del Valle, Cali, Valle del Cauca, Colombia

**Keywords:** Technology in healthcare, Artificial intelligence in medicine, Covid 19 mitigation, Intense care units everywhere, mechanical ventilators, AWS implementations, Evalu@ implementations, Artificial Neuronal Networks in medicine

## Abstract

The recent Coronavirus disease 2019 (COVID-19) pandemic displayed weaknesses in the healthcare infrastructures worldwide and exposed a lack of specialized personnel to cover the demands of a massive calamity. We have developed a portable ventilator that uses real-time vitals read from the patient to estimate -- through artificial intelligence -- the optimal operation point. The ventilator has redundant telecommunication capabilities; therefore, the remote assistance model can protect specialists and relatives from highly contagious agents. Additionally, we have designed a system that automatically publishes information in a proprietary cloud centralizer to keep physicians and relatives informed. The system was tested in a residential last-mile connection, and transaction times below the second were registered. The timing scheme allows us to operate up to 200 devices concurrently on these lowest-specification transmission control protocol/internet protocol (TCP/IP) services, promptly transmitting data for online processing and reporting. The ventilator is a proof of concept of automation that has behavioral and cognitive inputs to cheaply, yet reliably, extend the installed capacity of the healthcare systems and multiply the response of the skilled medical personnel to cover high-demanding scenarios and improve service quality.

## Introduction

Currently, the world population is passing through one of the most significant viral outbreaks in the modern era. The infectious agent, severe acute respiratory syndrome Coronavirus 2 (SARS-CoV-2), spreads fast and uses humans as vectors, a threat to our kind never seen before. ^1^

SARS-CoV-2 produces Coronavirus disease 2019 (COVID-19), a health condition characterized by respiratory infections. The level of affliction ranges from simple cold symptoms to severe illnesses that lead to general failure and death. ^2^

The Centers for Disease Control and Prevention (CDC) reported 624,088,072 confirmed cases and 6,552,725 deaths caused by COVID-19 worldwide by October 4th, 2022. ^3^

Approximately 80% of the world’s positive cases for COVID-19 recover from the disease without medical treatment. ^4^ The other 20%, including young individuals without pre-existing conditions, the elderly, and people with chronic illnesses, might present a severe affliction that could compromise the immune system and organs such as the lungs, heart, kidneys, liver, and brain. ^5^^,^^6^

The rapid spreadability of SARS-CoV-2 made contamination intensify over time, with exponential growth trending. ^7^ No country or health system in the world proved to have the infrastructure, resources, and response capacity to attend to the demand for care during contagion peaks. ^8^ With the intense care units (ICU) filled and medical personnel exhausted, any bed turned into a potential care unit; however, ventilators and specialized professionals are more challenging to find. ^9^ The scarcity of ventilators and specialized personnel repeated with every peak suffered worldwide.

When developing this work, South Korea, Vietnam, and Germany passed through a new peak of contamination with more than 200,000 new cases per day and more than 70 causalities in the same period, even when the cited countries reported vaccination done to 100% of the population. ^10^

This research reviewed mechanical setups, programmable electronics, embedded telecommunications, application programming interface (API) services, artificial intelligence (AI) implementations, and human respiratory variables to build a low-cost and highly reliable mechanical ventilator (MV). After reading the sensors, the device integrates a neuronal network capable of making control decisions in a closed loop that modifies the range and operating frequency of the device.

Additionally, the device reports the readings and control actions wirelessly to a data centralizer that can display the variables in real-time to any screen – including cell phones – lessening the need for dedicated and specialized personnel working in close contact with the infectious patients.

## Methods

This section describes the construction of a non-invasive MV controlled by a feedback artificial neural network (ANN). The developed MV reads the patient’s heart rate, oxygenation percentage, and respiratory pressure; and dynamically defines the working frequency of the ventilator and, consequently, the amount of oxygen to deliver.

Since low-cost sensors are involved in the MV’s operation, we trained an ANN with preconceived ‘true’ data to correct the outputs; therefore, the ANN makes the right decisions with an acceptable low rate of uncertainty regarding oxygen supply. The ventilator and the ANN are connected wirelessly within an AD-HOC network in a star topology that facilitates data transferring between a theoretically unlimited number of nodes. In addition, the effort to link new devices is low compared with firmware-based protocols such as the message queuing telemetry transport (MQTT). ^11^ Since the system uses the transmission control protocol/internet protocol (TCP/IP) protocol, the number of available nodes depends on the IP address class, the most common being the C class with 254 available nodes (assuming no subnetting). Recall that TCP/IP uses 32 bits logic addresses classified in A, B, C, D and E depending on how many octets are employed for allocating hosts and their purpose. Class C addresses are the ones with less hosting allocation capabilities and give Internet access to household residences. Since C class addresses reserve one octet for hosts, the current residential infrastructure can allocate *nd* = 254 devices ( *nd* = 2 ^8^ − 2).

### Ventilator’s general description

**Figure 1.**
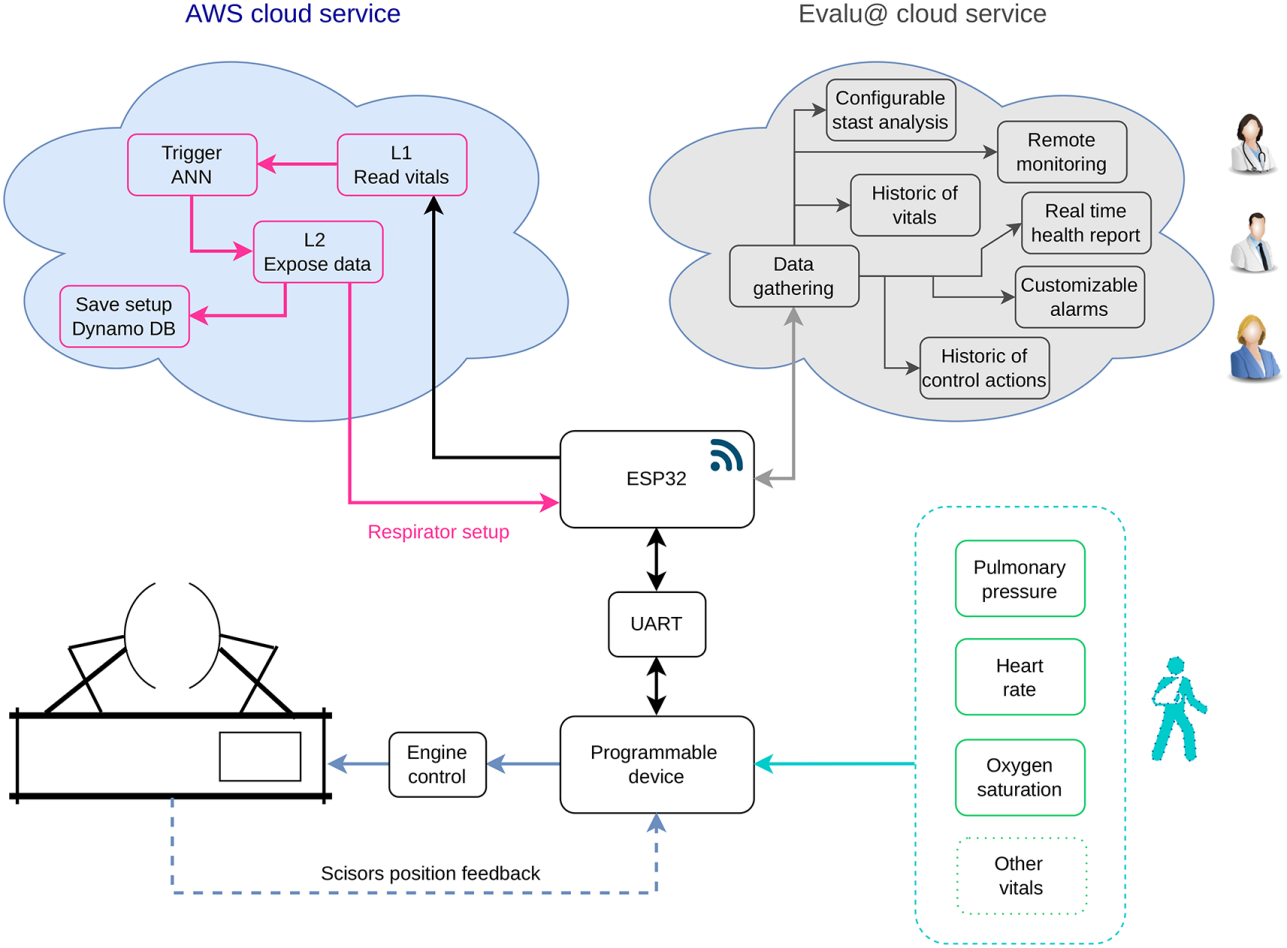
General diagram of the mechanical ventilator **.**  The system has access to Amazon Web Service (AWS) for on-demand intelligent setup and Evalu@ for warning and reporting purposes to the side of the specialists and patient's relatives. ANN, UART, DB, and L# stand for artificial neuronal network, universal asynchronous receiver-transmitter, Database, and Lambda specification in AWS, respectively.

A programmable device like BeagleBone Black (BBB) is the local brain of the system, synchronizing the sensors (inputs) and the actuator (output). This local brain interchanges information with the Amazon Web Service (AWS)[ 1] that allocates the ANN and returns operating configurations. The BBB and the AWS use an ESP32 chip as a data gateway with a universal asynchronous receiver transmitter (UART) to connect the BBB and TCP/IP on the side of the ANN. Using a homemade API, the gateway also pushes the estimated operation points, and the patient’s vitals to the Evalu@ service. ^12^ Since medical applications do not admit information lost (insufficient information or delayed data due to packets lost will affect the decision stage and eventually the patient’s health), we sacrificed speed and preferred reliability by choosing TCP instead of the user datagram protocol (UDP). Recall that UDP is a faster protocol with no recovery mechanisms in case information packets are corrupted or lost. ^13^ The firmware is written in C, ensuring faster operation than other programming platforms. ^14^ See a general diagram of the AI-based ventilator in Figure 1.

### Device mechanics and interfaces

**Figure 2.**
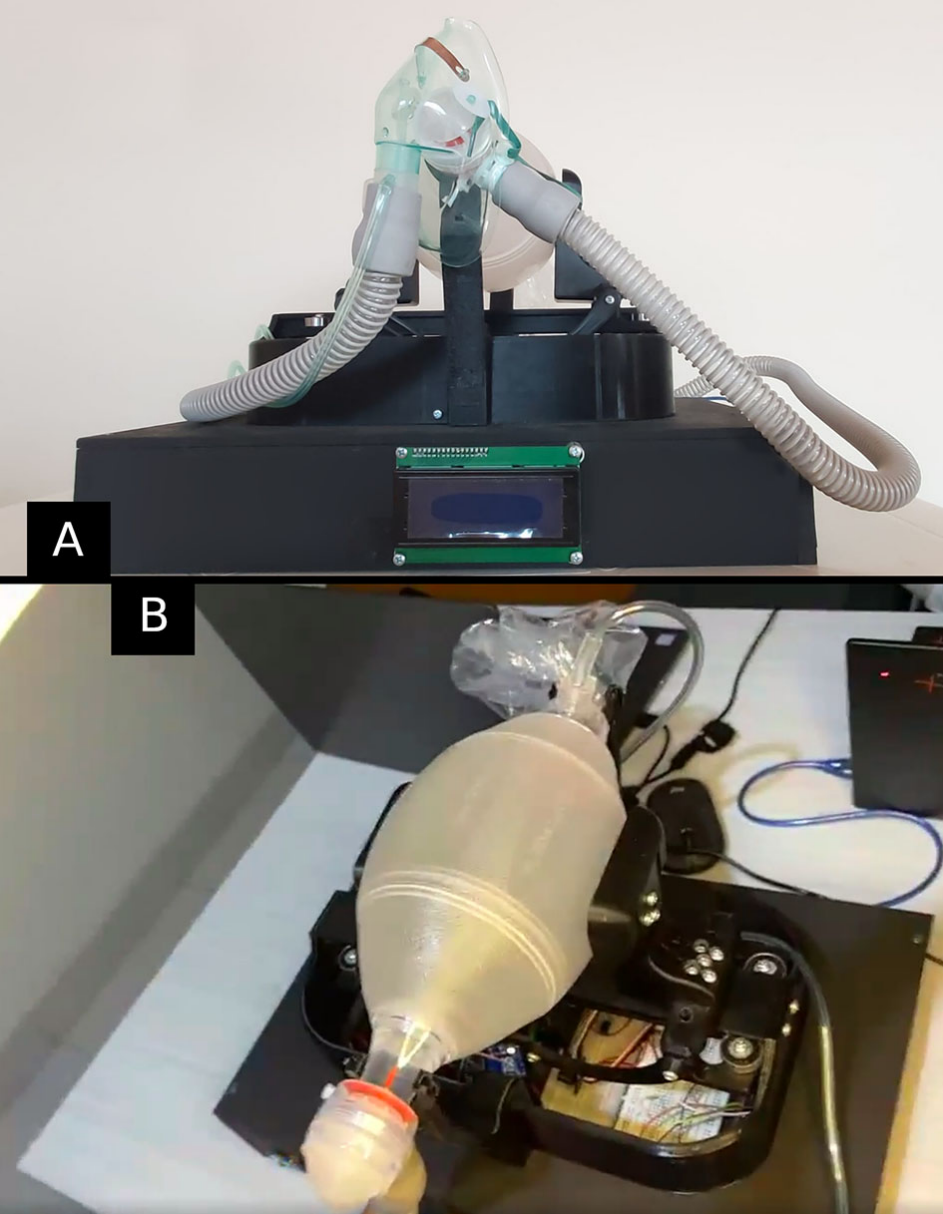
Front (panel A) and superior (panel B) views of the mechanical ventilator.

We designed the core structure of the ventilator with a plastic resin to provide resistance to the mobile parts while making the device portable and durable. A servomotor connected to the scissors-like levers activates the degree of freedom associated with the scissors’ opening angle that ultimately presses a flexible container. A predefined rotation setup exerted by the servomotor pushes the needed air to the pumping container, thus, to the patient. The system controls the air’s volume and delivery frequency with this arrangement. The ventilator has a buzzer, lighting, and a 16x2 matrix screen to account for in-site alarms and state displaying. See the ventilator’s physical appearance in Figure 2.

### Device’s Sensors

The MPX2010 differential pressure sensor, developed by Freescale semiconductors ®, has a linear transfer function *f* = 2.5/1[ *mV*/ *KPa*], and an output voltage span of [0-25 mV]. ^15^ The sensor yields a pressure value ( *P _i_*) by subtracting the input ( *P*1 *_i_*) – connected indirectly through the pumping system to the patient’s airways – from the value exerted in the vacuum input ( *P*2 *_i_*). An adjusting factor *Af* = 0.17 affects every *P _i_* in the P array to translate the readings from KPa to MBAR units (See **Equation** 1).

$$P_{i}=\left(P1_{i}-P2_{i}\right)*\mathrm{Af}\;\forall \;i\;\in \;P$$ (1)

The patient’s pulmonary pressure is calculated from the maximum values in the *P* array.

Regarding the heart rate (HR) and oxygen saturation, the MAX30100 chip developed by Maxim Integrated ®, reads both variables. It uses a visible red light (660 nm) and a receptor to capture intensity reflections after hemoglobin changes due to heart pulsation. ^16^ As for the oxygen saturation, the chip implements a second source of light working at the near-infrared spectrum (880 nm). The module determines the absorption spectrum of oxygenated and deoxygenated hemoglobin with the two light sources that produce interchange reflection peaks at the respective wavelengths. The MAX30100 digitizes the reflections of both light beams with a resolution of *V cc*/2 ^16^ and returns the sensed variables in human-readable format through the Inter-Integrated Circuit (I2C) interface.

### Artificial neural network construction

**Figure 3.**
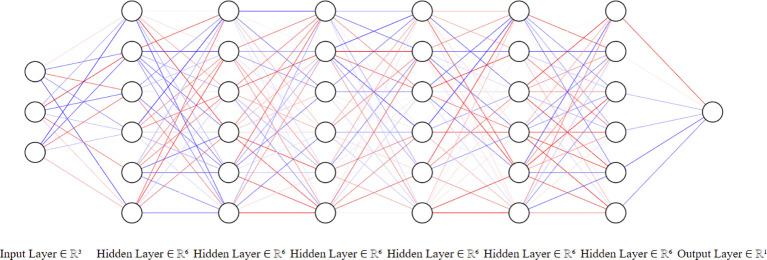
Artificial neural network (ANN) architecture. The initial layer receives the three vitals of the patient, and the output node returns the operation point that controls the amount of delivered oxygen.

We designed a six-layered ANN (See Figure 3). The input layer has three nodes to receive the heart rate, oxygenation percentage, and respiratory pressure. The output layer consists of one node, which provides the dynamic operation setup for the servomotor and ultimately controls the oxygen yield to the patient. The training data consisting of 1000 formulations with the three used features, and the supervising variable is available online here. ^17^ The training data was gathered from ICU records performed by Instituto de Genetica (Genesis S.A.S). The data is fully anonymized and respect the health insurance portability and accountability act (HIPAA) ^18^ directives.

The ANN uses two activation functions, namely Sigmoid and Identity, consequently, the system has a dynamic input/output ratio. The activation functions simulate the evoked potential that controls the release of neurotransmitters within the neurons in alive subjects. In the ANN context, the identity function is a mathematical activation model governed by the expression *f*( *x*) = *x*; therefore, it transfers the input to the output in a range ( ∞ , ∞ ). The sigmoid or logistic activation function, also known as a binary transfer, is governed by the expression $f\left(x\right)=\frac{1}{1+e^{-x}}$ and its range is (0, 1). The training process consists in adjusting the nodes’ outputs, so the combined work of the network yields numbers close to the supervising values. Backpropagation with a controlled gradient descent was implemented to modify the weights in the internal ANN layers while searching for optimization. The controlled gradient descent is accomplished using the Levenberg-Marquard strategy (trainlm) ^19^ that converges fast to optimization even when a wrong initial optimization guess is selected and provides mechanisms to avoid oscillations around the optimal value. The trainlm, is a distance minimization algorithm that receives an initial guess from the user and interactively updates a variable *β.* The method is fully implemented in python and is available in https://github.com/jjhartmann/Levenberg-Marquardt-Algorithm

### Data collection and integration

Sharing and processing the sensor data between the ESP32 and the AWS was accomplished through the lambda specification. ^20^ We start by provisioning a Virtual Private Cloud (VPC) ^21^ with the necessary utilities to connect and display the reports of the services to be consumed. Within the VPC architecture, one lambda triggers the microservice to establish the connection and feed the ANN with the three patient vitals. A second lambda triggers the microservice to obtain the ventilator setup yield by the ANN, which is delivered to the device’s hardware.

### Data storage

Patients’ data and actions taken by the ANN are sent to the Evalu@ service for storing, querying, analysis and reporting. This centralizer allows custom reports creation and online analysis through intuitive Excel templates, providing the flexibility to produce, in real-time, material to healthcare providers and relatives of the patients as well. A copy of the control values yielded by the ANN is also stored in the Dynamo DB service ^22^ of the AWS.

### Testing process

After establishing the ANN architecture, we ran a standard validation process by comparing the supervisor factors with the outputs yielded by the ANN. For the validation, we performed a three-folded exercise using 30% on the training data referred to in section *Artificial neural network construction.* We randomly selected the data for each folding. We obtained a 95.79% accuracy during these testing sessions using a Sigmoid activation function. The accuracy index decreased to 94.01% when using the Rectified Linear Unit (ReLU), ^23^ and the best performance regarding accuracy appeared when using a combination of Sigmoid and Identity functions among the ANN layers.

### Evalu@ configuration

**Figure 4.**
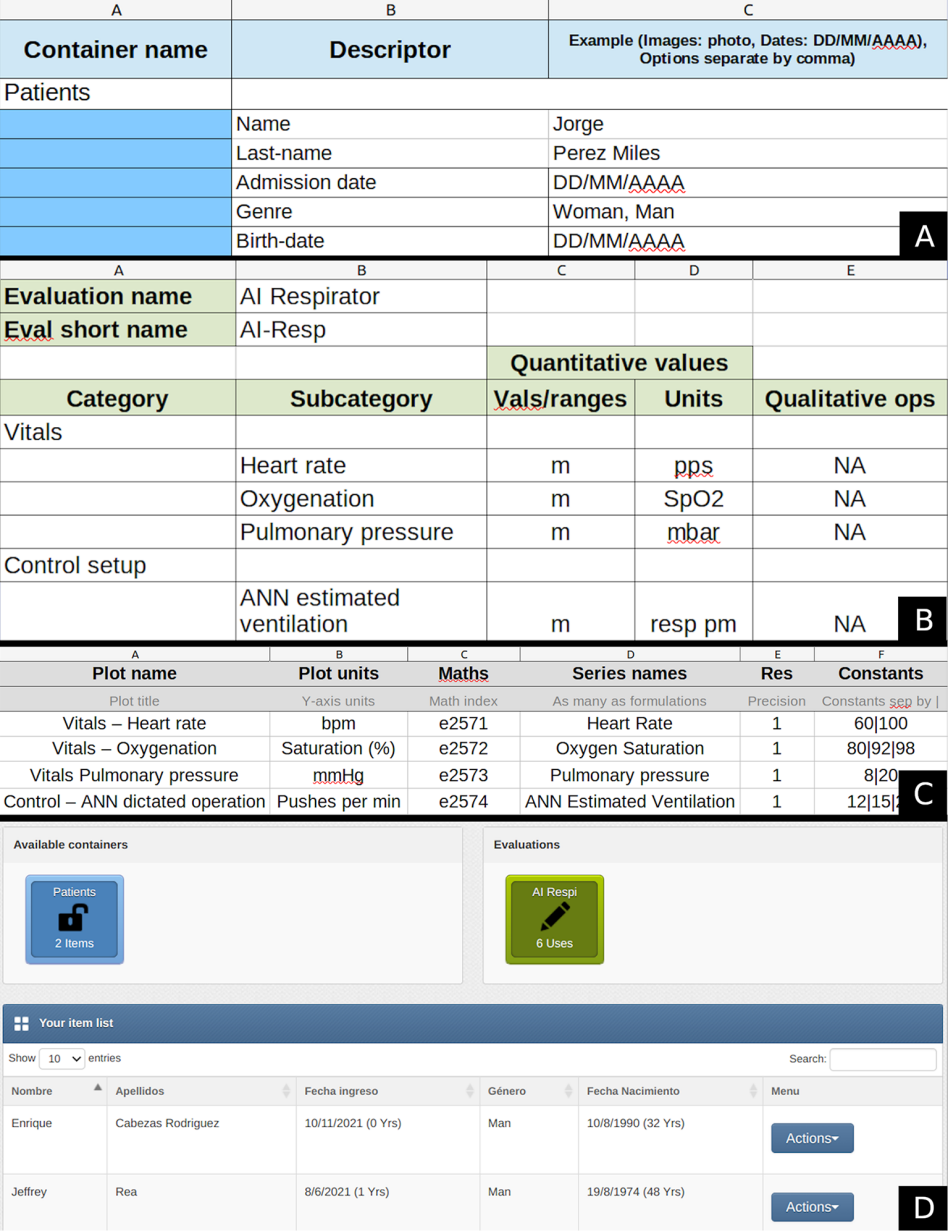
Evalu@ configuration. Panels A, B, and C show the configuration files used to create the containers, the tracking instruments, and the indexes’ visualization, respectively. Panel D displays the main screen after setup completion.

The Evalu@ service ( here) provides an intuitive mechanism to configure any tracking/monitoring scheme. The platform interprets the entries of three editable Excel files to create containers for the items to be evaluated, the tracking instruments, and the analysis that should be performed with the gathered data. Figure 4 presents the configuration files for the current application and Evalu@’s starting interface once the setup is finished.

Evalu@ is needed to present the data appealingly to users other than developers. It can also allocate the services executed now in AWS. However we leave this to further developments. Readers can now reproduce our methods using AWS and the provided code. If requiring the use of Evalu@, code C08 in the Zenodo repository displays the API to do so, and users testing the presented methods can use the Evalu@ service for free.

## Results

### ANN performance and solution timings

The Table 1 shows the performance of the python ANN implementation with the selected configuration, using a combination of Sigmoid and Identity functions among the ANN layers.

**Table 1.** Artificial neural network performance.

Operation	Accuracy (%)	Error Rate (%)
Fold 1	93.3	6.6
Fold 2	94.0	6.0
Fold 3	95.8	4.1

We ran data processing tests on the services exposed on the ESP32 (gateway) regarding the AWS and Evalu@ services. The records in Table 2 are the response times for one ventilator.

**Table 2.** Device's response times. The response times include query preparation, request delivery, and processing with the ventilator connected to a residential network with 20.14 and 12.23 Mbps download and upload speeds, respectively. The registered time is the average of five readings. A human request triggers the services labeled with a * in Evalu@.

Service	Action	Time (ms)
**AWS**	Send vitals	323.00
	Receive respirator setup	361.05
**Evalu@**	Send data (vitals, setup)	311.15
	Display data *	458.35
	Trigger alarm	124.74
	Report generation *	1065.12
	Setup alarms *	298.06

With the records in Table 2, we estimated the bursting capacity of the solution by simulating 200 concurrent users, adding to a total latency of 12 seconds. Recall that Internet connectivity in residences, where this development is intended to work, can allocate a maximum of 254 devices; however, since wifi performs like having the devices connected through a hub, latency can exponentially deteriorate when the technical limit of connections is reached.

### AWS records on a patient

**Figure 5.**
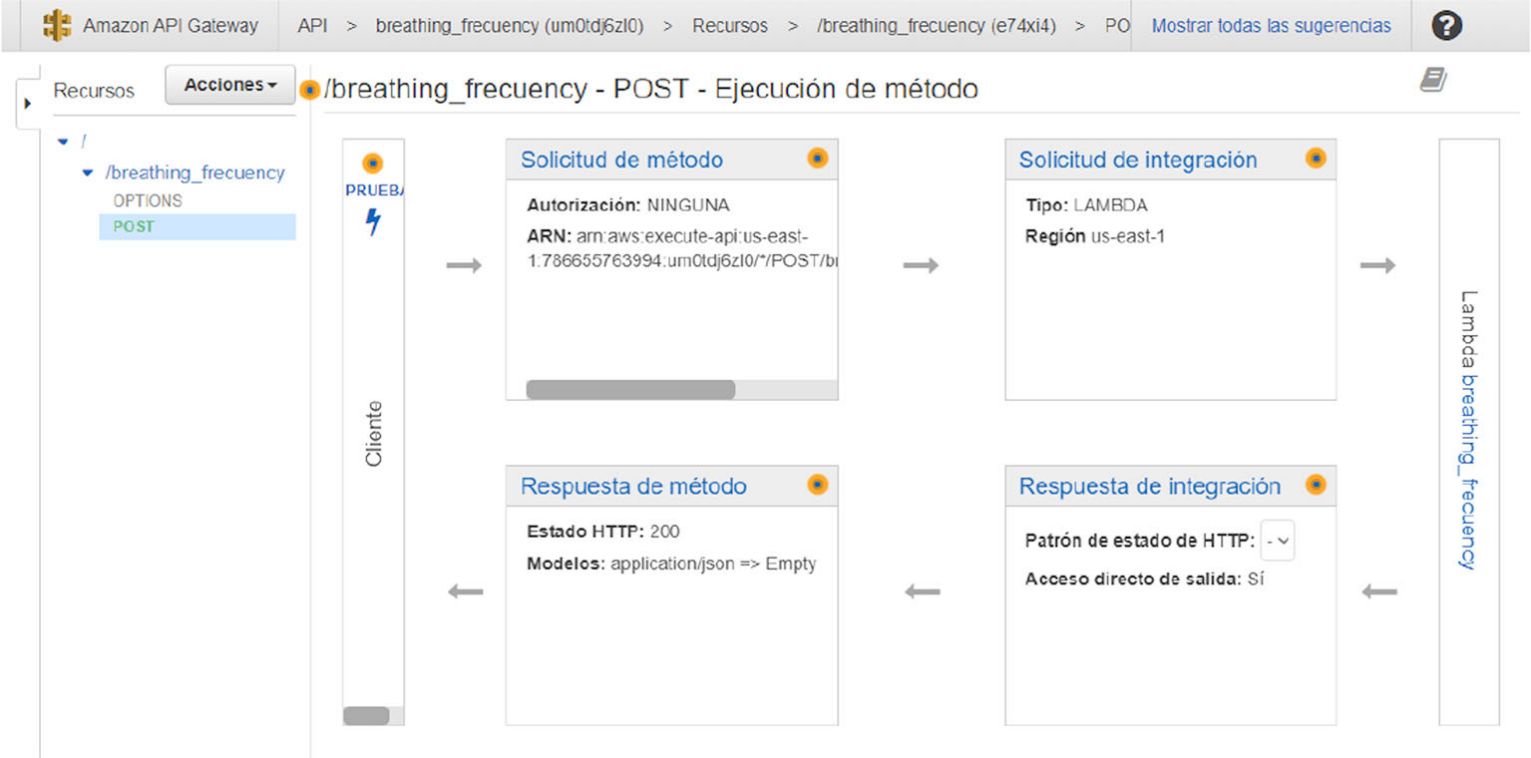
Architecture of the system from the Amazon Web Service perspective to yield the breathing frequency.

The VPC at AWS presents an interface to visualize the system’s architecture and implemented jobs. Figure 5 shows the AWS API gateway flow map for processing the breathing frequency.

**Figure 6.**
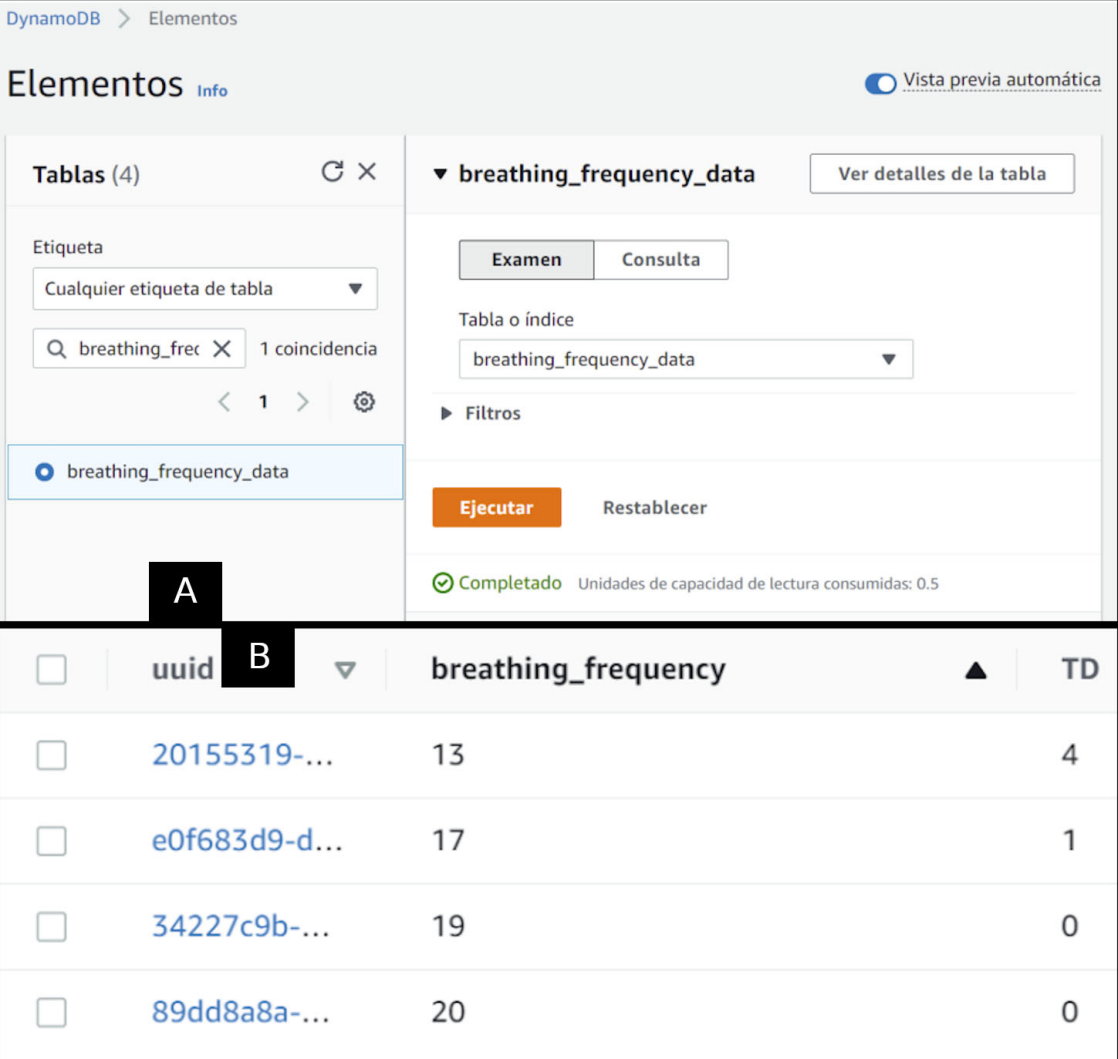
In panel A, the configuration of the table in the AWS Dynamo DB to store the estimated operation points. In Panel B, some operation point records.

The AWS produces control values to set the ventilator’s operation. We store the control setups produced by the ANN in the Dynamo DB as shown in Figure 6.

### Evalu@ records on healthy volunteer

**Figure 7.**
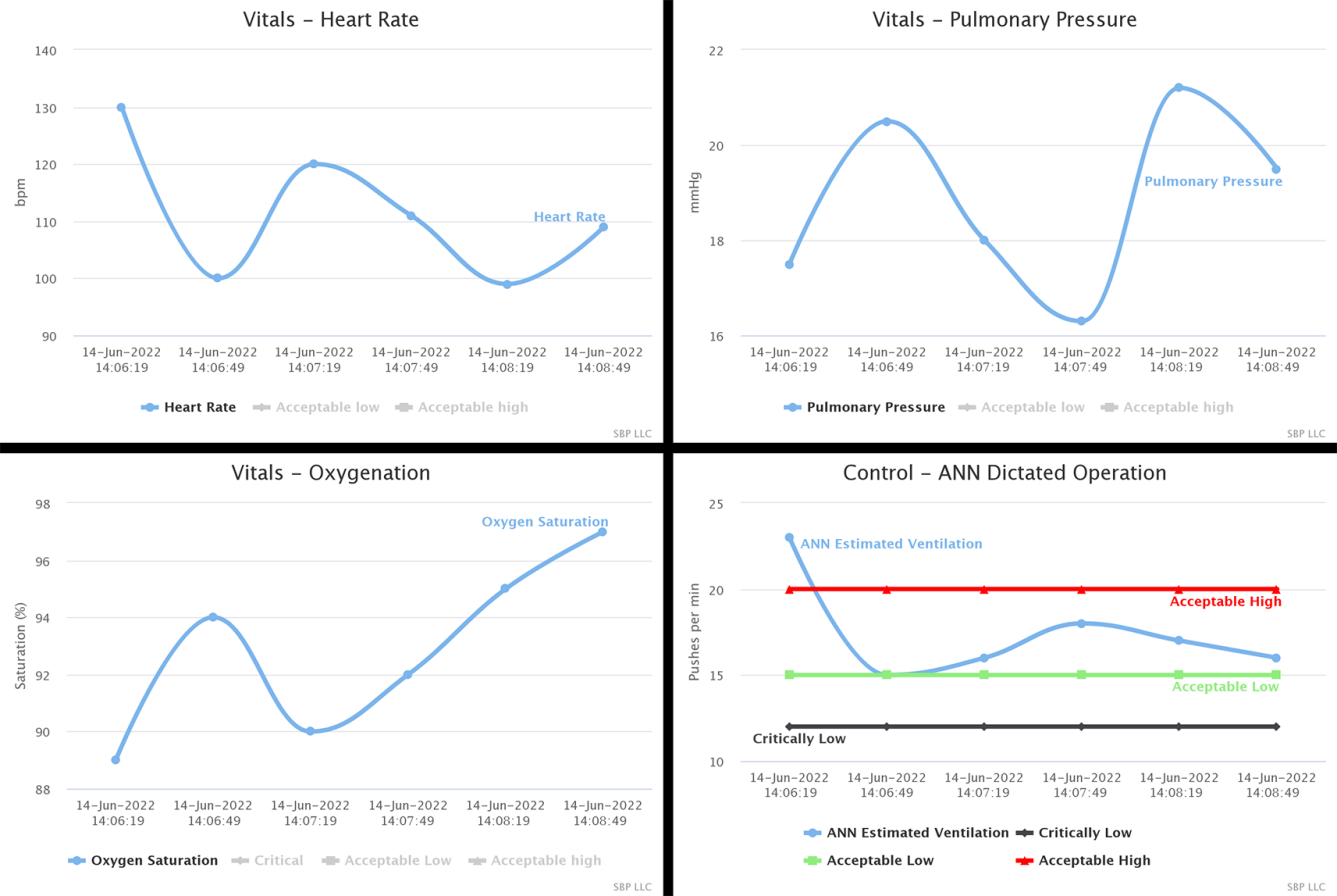
Evalu@ displaying vitals and the control signal in the timeline. The vitals are presented without alarm levels. The control signal has all the levels activated. It generates mail alarms – in addition to those generated in-site – when the control signal is above or below the established levels.

Although AWS can intuitively present the generated information, their displaying schemes are intended for development and debugging. Not to mention the commercial strategy that charges the user after reaching an established quota. Instead, Evalu@ has mechanisms to present the data to non-specialized users. We employ Evalu@ to present physicians and patients’ relatives with real-time information on vitals and control variables (see Figure 7).

### Ventilator operation without exceptions

**Figure 8.**
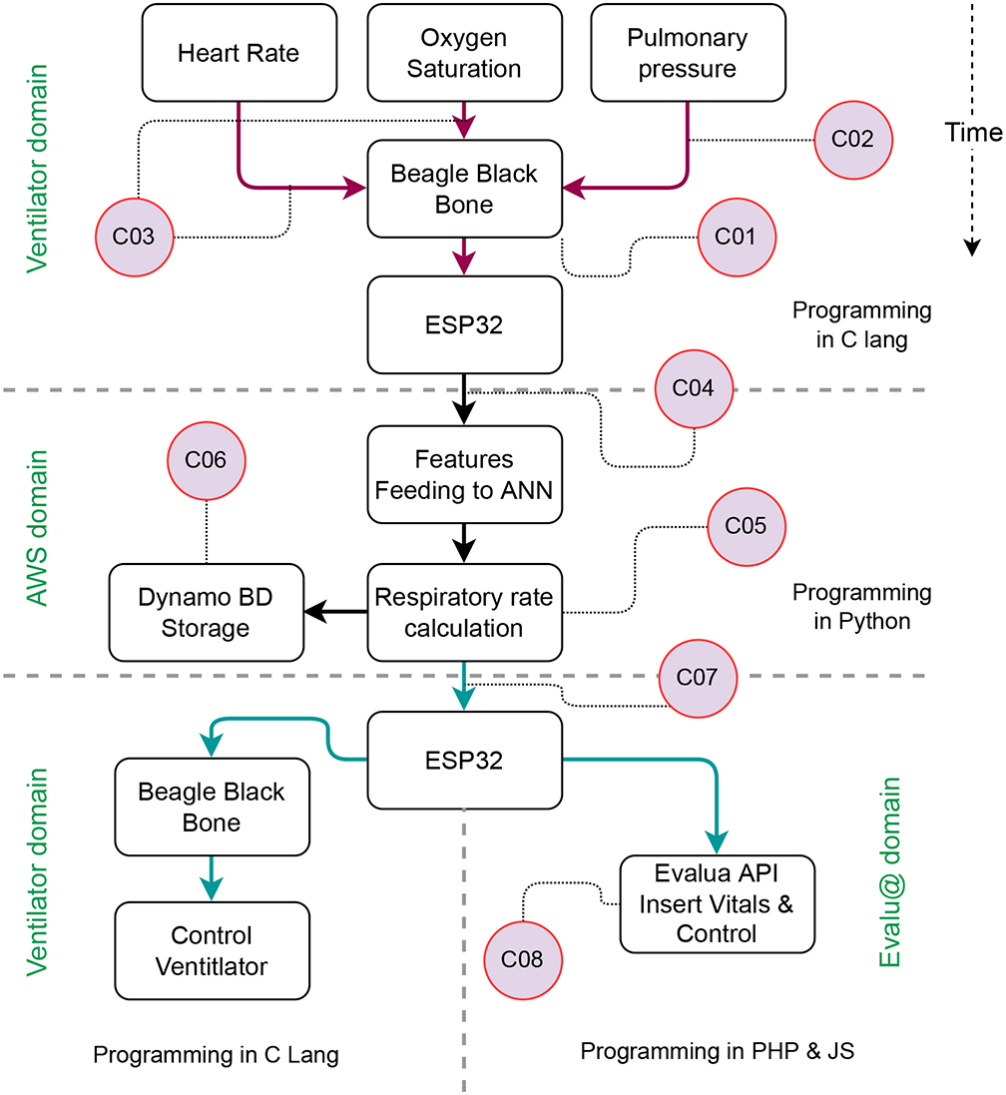
One loop run for a single ventilator. The rounded symbols refer to code supporting the functionality that has been presented as complementary material to assert reproducibility.

The Figure 8 resumes one loop operation of the system and provides step-by-step linking to the code deposited in https://doi.org/10.5281/zenodo.7400986. The system starts by configuring the ports and screen (C01). Then, it will read and process the patient’s vitals (C02-C03). The ventilator sends the vitals to the AWS using the ESP32 and the API (C04). The AWS uses the lambda specification to feed the ANN with the vitals and recovers the breathing-frequency variable (C05), which is saved in the DynamoDB (C06). The ESP consumes the API (C07) to recover the breathing frequency used by the ventilator to update the duty cycle of the Ambu system. The ESP also sends the vitals and control variables to the Evalu@ service using the API presented in C08. The authors encourage using the AWS S3 service, which will be enough to reproduce the results presented in this document. If an interface is needed to show the results to non-technical users, the authors warrant using Evalu@ at no cost for this particular application.

## Discussion

During the SARS-CoV-2 outbreak and due to the high demand for mechanical respirators, developers responded with prototypes intended for intensive care unit (ICU) use. ^24^^,^^25^ Additionally, medical personnel worked extended hours to cover the high demand for specialists and exposed themselves to the viral agent. ^26^ Companies and independent developers rushed to build prototypes and devices to cover the primary necessity at the cost of the high demand. However, the shortage was not only in materials but in personnel.

The presented design aims to assist patients in any place where one can improvise a bed while accounting for the shortage of specialized personnel by providing the systems with autonomous decision capabilities based on artificial intelligence. We also propose using *ad hoc.* networks to maintain distance with aerial and rapid transmission agents. The proposed strategy does not exclude the healthcare specialists; instead, it suggests that a cooperative environment employing highly available systems with configurable alarms will enhance the working environment leading to higher productivity while protecting healthcare personnel from aerial pathogens.

The created ventilator exploits telemedicine and allows health professionals to assist several patients with a glance at the reporting screen provided by Evalu@ with worldwide coverage, authenticated access, and adherence to HIPAA regulations. ^18^

The developed device can make autonomous decisions in real time. Professionals can attend to a larger group of people by monitoring their vital signs and prioritizing patients with more severe complications. In addition, the device can trigger alarms by value or trend so that care is no longer dedicated but demand-driven. Such a strategy positively impacts relevant aspects like efficiency and operating costs.

The proposed architecture uses standard telecommunications protocols, such as the TCP over WiFi (wireless fidelity) or GSM (global system for mobile communication) networks, assuring message integrity. Moreover, the SigFox ^27^ protocol can be implemented as we did in a previous research-transferred development that reached commercialization ^28^ to warrant operation in rural zones.

The implemented design is not rigid; therefore, we can add more sensors, and both the AWS and Evalu@ have the flexibility to afford the increase in processing and workload.

We are currently working on transferring the whole ANN processing to Evalu@ since the platform belongs to SBP Research and the research team led by author FYC. Evalu@ presents advantages regarding usability without incurring high costs derived from the expected massive use and long-term operation.

This development complements the devices created as proofs of concept supporting Patent No US20200273551A1. ^29^ The patent claims healthcare can move from a curative/preventive perspective to a predictive scheme where we can anticipate maladies occurrences using AI, which increases survival rates while making more efficient use of healthcare funds. The patent protects the design of an architecture that enables massive data gathering intended for artificial intelligence implementations. How the data is produced – algorithms and feeding devices – is out of the patent’s scope.

## Conclusions

In the apparent decline of the most recent pandemic, humanity learned – the hard way – several crucial lessons. We know now that pathogens can turn off worldwide activities, kill massively, and use humans as infectious vectors; a combination of factors compromising the existing infrastructure and rendering resources insufficient. The impact of further attacks would depend on how we optimize the resources. The inclusion of technology has the potential to place goods and means everywhere. Additionally, AI enables the reproduction of cognitive skills at a low budget to face difficulties with enhanced capabilities and be more efficient in calmed days. The presented ventilator is a proof of concept to demonstrate the feasibility of distributed healthcare that is assisted by reasonably cheap technology. It also automatically gathers reliable data that progressively empowers AI to derive verdicts and increase the accuracy of automated decisions.

## Ethics and consent

The patient data presented is taken from an author who volunteered to test the equipment presented. Ethical approval was not sought out for this study as it is considered to be of low risk and is not an intrusive test. There were no drugs or contrast agent administered and the author was awake at all times.

## Data Availability

Zenodo: Human Respiration Dataset - Training purposes https://doi.org/10.5281/zenodo.7324274. ^17^ This project contains the following underlying data: patientA_30secs.csv holding the records of vitals read on a healthy individual and control setups for a 5-minutes run. trainingDS.csv is a 1000 formulations file listing the three features and the supervising factor used during training and validation of the ANN. Data are available under the terms of the Creative Commons Attribution 4.0 International license (CC-BY 4.0).

## Footnotes

^1^
S3 specification - free service until 5G/Year with AI plugins can be found here

## Software availability

**Archived source code at the time of publication:** https://doi.org/10.5281/zenodo.7400986. ^30^

Software are available under the terms of the Creative Commons Attribution 4.0 International license (CC-BY 4.0).

This project contains the following underlying code:

VentilatorCode.html

VentilatorCode.ipynb

Both files have the same content, but the one with the extension ipynb can be loaded in Jupyter-notebook or online in Colab IDE.
