# Supplementary material for: Turning any bed into an intensive care unit with the Internet of things and artificial intelligence technology. Presenting the enhanced mechanical ventilator
Source: F1000Res. 2022 Dec 23;11:1570. [Version 1] doi: 10.12688/f1000research.127647.1 (PMC9925877; doi:10.12688/f1000research.127647.1)
Supplement: Peer review history

# Peer review: Reviewer response for version 1

**DOI:** 10.5256/f1000research.140177.r158686

**Published:** 2023-02-13

**Authors:** Maria Florencia Pollo-Cattaneo

**Affiliations:** 1 Information System Methodologies Research Group (Grupo GEMIS), Universidad Tecnológica Nacional Facultad Regional Buenos Aires, Buenos Aires, Argentina

This article proposes to provide a respiratory assistance service (portable mechanical ventilator) using technologies provided by artificial intelligence to optimize its operation. It includes the hardware and software architecture used, data management and interfaces.

 The problem is approached from the pandemic produced by COVID-19 and the hospital requirements in the face of the worldwide health crisis. The topic is relevant and of interest. The motivation is genuine and seeks to improve people's quality of life.

 The objective and methodology used is correct and consistent with the approach taken. Some sources of similar or previous works on the subject are not identified.

 The mechanical processes used and the ANNs used are described. 

 The results are promising and it is perceived that it is an advanced work.

 The conclusions are relevant. Future lines of work are still to be identified and if possible a medical evaluation by a professional specialist in the area should be available. 

 The following suggestions are made:

1. To revise the texts of some figures and their language. 
2. It is not clear the selection criteria of the chosen neural network topology. Nor why this technology is used.
3. A description of why AWS is used could be complemented.
4. To revise the texts of some figures and their language.
5. It is recommended to have a technical report describing the mechanics and construction of the system.
6. To consider in future lines scalability issues and possible conflicts (of connectivity and own of the development of the mechanics of the ventilator).

Is the rationale for developing the new method (or application) clearly explained?

Yes

Is the description of the method technically sound?

Yes

Are the conclusions about the method and its performance adequately supported by the findings presented in the article?

Yes

If any results are presented, are all the source data underlying the results available to ensure full reproducibility?

Partly

Are sufficient details provided to allow replication of the method development and its use by others?

Partly

Reviewer Expertise:

Information technology, artificial intelligence, machine learning, digital transformation

I confirm that I have read this submission and believe that I have an appropriate level of expertise to confirm that it is of an acceptable scientific standard.

# Peer review: Reviewer response for version 1

**DOI:** 10.5256/f1000research.140177.r158687

**Published:** 2023-01-25

**Authors:** Andres Felipe Montaño

**Affiliations:** 1 TECNALIA - Basque Research and Technology Alliance (BRTA), Gipuzkoa, Spain

This paper describes the hardware and software architectures of a portable mechanical ventilator (including data management and the user interface) that uses artificial intelligence to estimate its optimal operation point. It is motivated by the need to provide high quality service in high-demand scenarios, such as the recent COVID-19 outbreak.

 It measures the patient's heart rate, oxygen saturation, and pulmonary pressure as inputs. The sensors used to obtain this information are described. An artificial neural network (ANN) uses this information to estimate the breathing frequency. Using this parameter, mechanical arms are controlled around an Ambu bag to provide oxygen to the patient.

 In general, the motivation and objectives are relevant, and the paper is well written and easy to read. The introduction is very clear and explains very well the motivation of the work. This seems a mature work, result of integration of the experience of the author in related fields (hardware and software design).

 Some comments are as follows.

 Description of the system is clear, but the authors could provide more detailed information of the mechanical parts of the system.

 To enrich the content of the article, a discussion of the decisions in designing the neural network and the motivation for choosing an ANN could be added. Information provided in Figure 3 is not relevant for understanding the behavior or design discussion of the system. The ANN appears to be a critical component of the system and the decision of implementing it in an external service such as AWS should be better supported. At least explain why it is not implemented locally, leaving only the database and the display information to the external services provided by the internet (through AWS and Evalu@). In my opinion, an AI expert should review the article.

 There should be a more detailed comparison with previous/similar works (including in the references but not remarked/compared with the proposed system).

 Please, review/evaluate the content of Figures 5 and 6, if they are considered relevant update labels from Spanish to English, but, for example, part B of figure 6 does not seem to add information to the paper.

Is the rationale for developing the new method (or application) clearly explained?

Yes

Is the description of the method technically sound?

Yes

Are the conclusions about the method and its performance adequately supported by the findings presented in the article?

Partly

If any results are presented, are all the source data underlying the results available to ensure full reproducibility?

Partly

Are sufficient details provided to allow replication of the method development and its use by others?

Yes

Reviewer Expertise:

Electronic system, Robotics, software design

I confirm that I have read this submission and believe that I have an appropriate level of expertise to confirm that it is of an acceptable scientific standard, however I have significant reservations, as outlined above.

# Peer review: Reviewer response for version 1

**DOI:** 10.5256/f1000research.140177.r158688

**Published:** 2023-01-06

**Authors:** Christian Grévisse

**Affiliations:** 1 University of Luxembourg, Esch-sur-Alzette, Luxembourg

The authors describe a portable ventilator to enable remote monitoring and handling of patients in contexts of contagious agents. The motivation behind this is to cover both lack in specialists and material in high-demand settings such as the COVID-19 pandemic. The device comprises sensors to measure a patient's heart rate, oxygen saturation and pulmonary pressure. These vital signs are given to an artificial neural network (ANN) which estimates the breathing frequency. This parameter then controls mechanical arms around an Ambu bag.

**AI:**

- The authors do not explain why an ANN has been chosen, compared to other machine learning methods. It would be helpful to justify this choice or even compare it.
- There is insufficient information on the training data, i.e., no indication about age, gender or pathologies. This also raises the question for which population (age, gender, pathologies) this ventilator can be used.

**Devices:**

- Are the sensors proven to provide accurate data (e.g., approval through the FDA), or were their data compared to clinical instruments?
- The paper provides little information on the home-made ventilator itself. The authors should provide reconstruction plans and overall indicate fees and sustainability aspects (e.g., materials, energy consumption).

**Networking:**

 Why is an ad-hoc Wifi network used? The explanation about maintaining distance with aerial and rapid transmission agents (second paragraph of the "Discussion" section) is not very sound. In any case, a connection to the Internet is required to establish connection to the two remote services (AWS and Evalu@). Please further explain the downsides of an infrastructure Wifi.

**Remote services:**

 As the data from the ANN is crucial for the operation of the ventilator, should the ANN not rather be run on a local device to avoid negative effects of network latency, congestion or unavailability?

**Privacy:**

- Did the authors consider privacy-related issues concerning the fact that patient data would be transferred to two remote services (AWS and Evalu@)?
- How (and by whom) is access to patient data on Evalu@ granted or revoked? What security measures are in place to protect this sensitive data?

**Testing and validation:**

- The paper mentions a volunteer, but there are no indications about their age, gender and health condition. The section "Evalu@ records on healthy volunteer" does not provide any information on the volunteer either, albeit mentioning them in the title.
- Has the device been tested on a high-fidelity manikin simulating different pathologies?
- Has the approach been validated by an emergency medicine specialist?

**General comments:**

- In the paragraph before the "Discussion" section, the authors mention about updating the duty cycle of the Ambu system. For audiences not familiar with emergency medicine, it would be beneficial to further explain this.
- In the first paragraph of the "Discussion" section, the authors briefly mention the prototypes developed during the high demand for respirators at the outbreak of the COVID-19 pandemic, yet fail to provide further details on similar approaches reported in the literature. It would be beneficial to compare other approaches to the setting proposed in this paper.
- Figures 5+6: Maybe redo these screenshots with English locale.
- Figure 8: Typo in the lower-left rectangle ("Control Ventitlator").
- Formatting issue in reference 17
- Conclusion: Do the authors have any plans for future work in this context?

**Code:**

- There is no code for controlling the ventilator after receiving the ventilation frequency from AWS.
- There is a syntax error in C05, second to last line.

Overall, the method proposed in this paper is promising, enabling a cooperative environment helpful for telemedicine settings during high-demand periods where lack of personnel can happen. However, during the beginning of the COVID-19 pandemic, the world faced a huge supply chain issue regarding silicon. As the proposed ventilator requires quite some electronic equipment, the lack of material could hamper the approach. What solutions do the authors see concerning this issue?

 The article should be further reviewed by an expert in AI methods as well as an emergency medicine specialist.

Is the rationale for developing the new method (or application) clearly explained?

Yes

Is the description of the method technically sound?

Yes

Are the conclusions about the method and its performance adequately supported by the findings presented in the article?

Partly

If any results are presented, are all the source data underlying the results available to ensure full reproducibility?

Partly

Are sufficient details provided to allow replication of the method development and its use by others?

Partly

Reviewer Expertise:

Computer science, e-learning applied to medical education

I confirm that I have read this submission and believe that I have an appropriate level of expertise to confirm that it is of an acceptable scientific standard, however I have significant reservations, as outlined above.
